# Neck Surgery for Non-Well Differentiated Thyroid Malignancies: Variations in Strategy According to Histopathology

**DOI:** 10.3390/cancers15041255

**Published:** 2023-02-16

**Authors:** Fernando López, Abir Al Ghuzlan, Mark Zafereo, Vincent Vander Poorten, K. Thomas Robbins, Marc Hamoir, Iain J. Nixon, Ralph P. Tufano, Gregory Randolph, Pia Pace-Asciak, Peter Angelos, Andrés Coca-Pelaz, Avi Khafif, Ohad Ronen, Juan Pablo Rodrigo, Álvaro Sanabria, Carsten E. Palme, Antti A. Mäkitie, Luiz P. Kowalski, Alessandra Rinaldo, Alfio Ferlito

**Affiliations:** 1 Head and Neck Surgery, Hospital Universitario Central de Asturias (HUCA), 33011 Oviedo, Spain; 2 Instituto Universitario de Oncología del Principado de Asturias, Instituto de Investigación Sanitaria del Principado de Asturias, University of Oviedo, CIBERONC, 330011 Oviedo, Spain; 3 Department of Biology and Pathology, Gustave Roussy Cancer Campus, University Paris-Saclay, 91190 Villejuif, France; 4 Department of Head and Neck Surgery, The University of Texas M. D. Anderson Cancer Center, Houston, TX 77030, USA; 5 Otorhinolaryngology Head and Neck Surgery and Department of Oncology, Section Head and Neck Oncology, University Hospitals Leuven, 3000 Leuven, Belgium; 6 European Reference Network for Rare Adult Solid Cancers (EURACAN), 69008 Lyon, France; 7 Department of Otolaryngology-Head and Neck Surgery, Southern Illinois University School of Medicine, Springfield, IL 62702, USA; 8 Department of Head and Neck Surgery, UC Louvain, St Luc University Hospital and King Albert II Cancer Institute, 1200 Brussels, Belgium; 9 Department of Otorhinolaryngology Head and Neck Surgery, NHS Lothian, Edinburgh EH1 3EG, UK; 10 FPG Thyroid and Parathyroid Center, Division of Head and Neck Endocrine Surgery, The Sarasota Memorial Health Care System, Sarasota, FL 34239, USA; 11 Division of Otolaryngology-Endocrine Head and Neck Surgery, Massachusetts Eye and Ear Infirmary, Harvard University, Boston, MA 02138, USA; 12 Department of Otolaryngology-Head and Neck Surgery, University of Toronto, Toronto, ON M5S, Canada; 13 Department of Surgery and MacLean Center for Clinical Medical Ethics, The University of Chicago, Chicago, IL 60637, USA; 14 A.R.M. Center of Otolaryngology-Head and Neck Surgery, Assuta Medical Center, Affiliated with Ben-Gurion University of the Negev, Tel Aviv 8410501, Israel; 15 Department of Otolaryngology—Head and Neck Surgery, Galilee Medical Center, Affiliated with Azrieli Faculty of Medicine, Bar Ilan University, Safed 5290002, Israel; 16 Department of Surgery, Universidad de Antioquia, CEXCA Centro de Excelencia en Enfermedades de Cabeza y Cuello, Medellín 050021, Colombia; 17 Department of Head and Neck Surgery, Sydney Head and Neck Cancer Institute, Chris O’Brien Lifehouse, Sydney, NSW 2050, Australia; 18 Faculty of Medicine and Health Sciences, Sydney Medical School, The University of Sydney, Sydney, NSW 2006, Australia; 19 Royal Prince Alfred Institute of Academic Surgery, Sydney Local Health District, Sydney, NSW 2050, Australia; 20 Department of Otorhinolaryngology-Head and Neck Surgery, University of Helsinki and Helsinki University Hospital, FI-00029 Helsinki, Finland; 21 Department of Head and Neck Surgery, University of Sao Paulo Medical School, Sao Paulo 01246-903, Brazil; 22 Department of Head and Neck Surgery and Otorhinolaryngology, AC Camargo Cancer Center, Sao Paulo 01509-001, Brazil; 23 School of Medicine, University of Udine, 33100 Udine, Italy; 24 Coordinator of International Head and Neck Scientific Group, 35125 Padua, Italy

**Keywords:** Thyroid cancer, non-well differentiated thyroid cancer, neck dissection, treatment

## Abstract

**Simple Summary:**

In non-well differentiated thyroid cancer, the rate of nodal involvement is variable and depends on the histology of the tumor. We aim to highlight the opinions of several experts from different parts of the world on the current management of the less common types of thyroid cancer to provide a consensus on the treatment of regional lymphatics for these entities. The rate of lymph node involvement is variable and depends on the histology of the tumor. Within undifferentiated tumors’, there is an established consensus on the treatment of anaplastic carcinoma, medullary carcinoma and poorly differentiated carcinoma of the thyroid. However, treatment of other rarer tumors must be individualized, taking into account both the aggressiveness of the histology and the anatomical distribution of the disease. In general, prophylactic treatment of the neck is not indicated.

**Abstract:**

Lymph node metastases in non-well differentiated thyroid cancer (non-WDTC) are common, both in the central compartment (levels VI and VII) and in the lateral neck (Levels II to V). Nodal metastases negatively affect prognosis and should be treated to maximize locoregional control while minimizing morbidity. In non-WDTC, the rate of nodal involvement is variable and depends on the histology of the tumor. For medullary thyroid carcinomas, poorly differentiated thyroid carcinomas, and anaplastic thyroid carcinomas, the high frequency of lymph node metastases makes central compartment dissection generally necessary. In mucoepidermoid carcinomas, malignant peripheral nerve sheath tumors, sarcomas, and malignant thyroid teratomas or thyroblastomas, central compartment dissection is less often necessary, as clinical lymphnode involvement is less common. We aim to summarize the medical literature and the opinions of several experts from different parts of the world on the current philosophy for managing the neck in less common types of thyroid cancer.

## 1. Introduction

Globally, thyroid cancer is the most common malignant endocrine tumor and accounts for more than 90% of the endocrine cancers [1]. Rather than thyroid cancer being a single disease entity, it is composed of different histologic types that originate from distinct embryological cells. Furthermore, there are several genetic mutations that distinguish one type from another. While well-differentiated thyroid carcinoma (WDTC) represents the majority of thyroid malignancies, the non-WDTC is a less common heterogenous group that portends an increased morbidity and mortality [2,3,4,5,6,7]. This diverse group includes poorly-differentiated thyroid carcinomas (PDTC), anaplastic thyroid carcinomas (ATC), medullary thyroid carcinomas (MTC), specific sarcomas, and primary thyroid teratomas. In addition, newer types have become recognized based on genetic mutations and embryologic cell-derived origins. Despite the rarity of these neoplasms, they remain an important part of the differential diagnosis for any thyroid mass. Under select circumstances, core needle biopsies for rapidly enlarging thyroid masses are now considered appropriate and expeditious [8,9].

The propensity for lymphatic metastases is variable based on the specific thyroid cancer type, the knowledge of which is an essential part of cancer treatment. It enables a more accurate staging and thus, a more precise estimation of prognosis, data-directed patient counseling, tailored treatment planning, and a more efficient follow-up. Generally, the presence of nodal disease tends to lead to a poorer long-term prognosis but varies depending on the histology of the cancer.

Not all thyroid cancers have the same predisposition for lymphatic metastasis. Within the broad spectrum of thyroid carcinoma, even within WDTC, there is the typical different mode of metastasis when comparing follicular thyroid carcinoma (FTC) (mainly hematogeneous route of metastasis) [10] to papillary thyroid carcinoma (PTC). PTC may or may not involve extension to the central compartment (level VI-VII) lymph nodes (such as prelaryngeal lymph nodes, pretracheal, and paratracheal lymph nodes) or lateral lymph nodes (most commonly levels II to IV) [11]. Also, some non-WDTC such as MTC [12] or PDTC are marked by a high susceptibility to metastasize to regional lymph nodes [13].

In this review, we aim to summarize the medical literature and views of several experts from different parts of the world about the current data on lymphatic drainage pathways of the less common thyroid cancer types, discussing non-WDTC (including PDTC, ATC, MTC, mucoepidermoid carcinoma (MEC), malignant peripheral nerve sheath tumors (PNSTs), angiosarcoma, leiomyosarcoma, and primary malignant thyroid teratoma or thyroblastoma) to provide a unified philosophy on surgical approaches to nodal disease in these entities. The lymphatic drainage characteristics of WDTC have been described elsewhere and will not be outlined in this review [1]. 

## 2. Lymphatic Drainage Pathways of the Thyroid Gland

Although the thyroid lymphatic network forms macroscopic structures, these are hard to visualize pre-operatively and intraoperatively without special techniques. Knowledge of the thyroid lymphatic drainage pathways is a fundamental part of cancer treatment. It enables a more accurate staging and assessment of prognosis, improved patient counseling, tailored treatment planning, and a more specific follow-up analysis.

The Italian anatomist Gaspare Aselli, in the early 17th century, takes credit for the first discovery and description of the lymphatic system [14]. Marcello Malpighi, Anton Nuck, and Marie Philibert Constant Sappey later used mercury injection to precisely demonstrate the delicate lymphatic pathways of the human body. Newer techniques using dyes and, more recently, indocyanine green dye with near-infrared fluorescence labeling enable visualizing these elusive structures more clearly. Historically, our understanding of lymphatic drainage and its embryonic origin has lagged, compared to other organs and systems. Recent discoveries in rats identified venous endothelial cells expressing the transcription factors Sox-18, and Prox1, which lead to the formation of early lymphatic vessels [15]. Recent animal studies strengthen this observation as lymphatic vessel development is dependent on prior angiogenesis [16]. Thus, in general, the lymphatic vessels usually follow the known blood vessels’ course. Furthermore, knowledge of blood vessels embryologic development enables the prediction of expected lymphatic drainage pathways.

The central compartment of the neck, which contains level VI-VII lymph nodes, is bounded superiorly by the hyoid bone, laterally by the carotid arteries, inferiorly by the sternal notch and brachiocephalic artery, anteriorly by the superficial layer of the cervical fascia, and posteriorly by the deep layer of the cervical fascia [17]. The central neck is subdivided into compartments that include lymph nodes in the prelaryngeal/precricoid space known as the ‘Delphian’ nodes, the pretracheal, and paratracheal nodes, all of which are important to be considered when surgically-resecting thyroid cancer [11,13,18]. The term ‘Delphian node’ (DN) was first suggested by Raymond V. Randall, inspired by the ‘Oracle of Delphi’, of 1400 BC Greece [13]. Metastatic involvement of DN is often associated with an isthmus primary thyroid cancer, extrathyroidal invasion, and a higher incidence of metastatic disease in the central and lateral compartments of the neck. When DN is positive on intraoperative frozen sections, careful evaluation of the central and lateral lymph node compartments is essential [13]. However, in practice, other than the DN, little meaningful lymph nodal tissue related to thyroid cancer exists above the level of the cricoid cartilage in the central compartment. Occasionally, there are small lymph nodes encountered adjacent to a long pyramidal lobe [19]. In a retrospective study by the Memorial Sloan Kettering group, 101 patients had their DN sent separately for pathology and reported that the median size of metastasis to this node is only 3 mm. This implies the possibility of it being inadvertently removed with excision of the pyramidal lobe or even during the separation of the strap muscles [13]. Nevertheless, its routine removal in any suspected thyroid cancer should be advocated. There is almost no risk to doing so routinely and recurrence/persistency here are more difficult to manage. Approximately 35% of patients presenting with WDTC have cN1 disease [20], but up to 80% of patients presenting with cN0 disease may harbor microscopically positive lymph nodes.

The lymphatic pathways also extend into the upper mediastinum, also designated as level VII, as well as laterally into level IV, in the trachea-esophageal groove [21]. While most of the medial anlage of the thyroid gland evolves and descends from the foramen caecum, the lateral anlage that gives rise to the upper poles and Zuckerkandl tubercle have a distinct embryologic origin [22]. For this reason, the thyroid superior pole lymphatics pathways can skip level VI first echelon nodes and drain directly to level II (upper jugular vein nodes) and level III (mid-jugular nodes) in up to 18% of patients without involvement of the central neck lymph nodes [21,23]. In a recent systematic review and meta-analysis, Zhao et al. [24] demonstrated that a PTC lesion situated in the superior gland has an increased risk of x3.6 to skip the central compartment and metastasize directly to the lateral neck. In a remarkable anatomical study, Sato et al. [25] meticulously dissected the thyroid region, revealing its intricate lymphatic drainage. Some lymphatic vessels pass through the paratracheal lymph node basin and continue posteriorly to the carotid sheath into level IV, whereas other lymphatic vessels pass anteriorly to the carotid sheath to lateral neck nodes including level Vb. Although skip-metastases occur in only a small number of patients, the lateral compartment should be carefully evaluated for metastases [23]. 

In an earlier study, Rouvière reported another lymphatic pathway of the thyroid gland to retropharyngeal nodes in 20% of cadavers. These are rarely observed but parapharyngeal and/or retropharyngeal lymph node metastasis from thyroid cancer are occasionally reported in primary tumors with extensive nodal disease or recurrences after previous neck dissection and where axial imaging beyond ultrasound alone is used to detect them [26,27,28].

## 3. Treatment of the Neck: Central and Lateral Neck Dissection

Among patients with thyroid cancer, it may be necessary to perform a neck dissection. A detailed description of the surgical procedure is beyond the scope of this publication and can be found in many publications [29].

If indicated, dissection of the central lymph node compartments (Level VI) should be performed at the same time as the primary thyroid surgery. It involves removal of lymphatic tissue from the prelaryngeal, pre- and paratracheal, and tracheoesophageal nodal basins [30,31]. During this dissection, special attention should be paid to the identification of the recurrent laryngeal nerves and parathyroid glands. The recurrent laryngeal nerves should be dissected with minimal traction. Of surgical relevance, the right and left paratracheal regions differ in terms of the lymph node distribution around the recurrent laryngeal nerve. To completely remove the lymph node compartments surgically, it is important to note that the lymph nodes in the right paratracheal compartment are both anterior and posterior to the right recurrent laryngeal nerve due to the presence of the brachiocephalic artery. For the dissection of the right paratracheal region to be complete, it is necessary to remove the lymphatic tissue deep to the right recurrent laryngeal nerve, for which the nerve must be transposed. This dorsal region deep to the right nerve is of specific concern because it is a common and unfavorable place for disease recurrence. On the left side, the lymph nodes are located anterior to the nerve because it courses posteriorly on the prevertebral fascia following recurring around the aortic arch (leaving no space for lymph nodes posteriorly) [20]. In order to achieve a complete surgical resection in the right paratracheal region, the RLN needs to be transposed to access the lymph nodes both anteriorly and posteriorly to the nerve [17]. In contrast, the lymph nodes in the left paratracheal region are located anterior to the RLN and the esophagus, which typically allows for less manipulation of the recurrent laryngeal nerve. Attention should also be paid to the parathyroid glands. In cases of suspected compromised vascular flow to the parathyroids, autotransplantation of these glands can be placed into a pocket within the sternomastoid muscle. The superior glands may be left in situ along with their blood supply. The inferior glands are more variable in position and are often required to be reflected laterally, attempting to maintain the vascular flow from the inferior thyroid artery.

The presence of metastatic disease in the lateral neck should be assessed pre-operatively. Ideally, neck surgery should be performed at the same time as the procedure for the primary disease. The goal of neck dissection is to remove all macroscopic lymph node metastases while preserving the neurovascular structures. The detection of nodal disease focuses on that which is “clinically apparent” and should be defined by pre-operative US and CT nodal mapping studies [32,33,34,35]. Although pre-operative assessment will guide the extent of resection required, levels II, III, IV, and Vb should be included in the lateral dissection [36]. In the absence of adenopathy involving levels IIa, III, and IV, metastases located cranial to the spinal accessory nerve (IIb and Va) or at level I are rare. Preservation of these areas will minimize the risk of injury to the marginal branch of the facial nerve and to the spinal accessory nerves, while preserving the submandibular salivary gland. The berry-picking operation should be avoided to prevent further lymph node recurrences, which will require further interventions [20].

## 4. Strategy According to Histology

### 4.1. Poorly-Differentiated Thyroid Carcinoma

**Figure 1:**
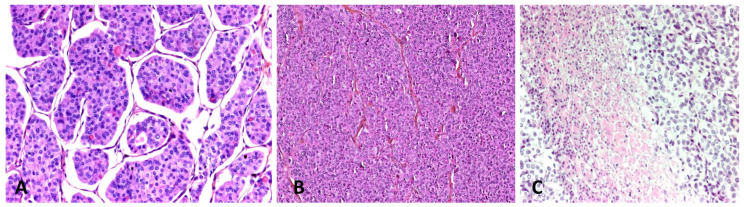
(**A**) PDTC with insular pattern (×200 magnification). (**B**) PDTC with solid pattern (×100 magnification). (**C**) PDTC area of necrosis (×200 magnification).

PDTC, according to Turin consensus, arises from follicular epithelial cells and accounts for less than 15% of malignant thyroid tumors [37,38] (Figure 1). PDTCs have an intermediate biological behavior between WDTC (papillary and follicular) and ATC. It can arise de novo or due to progression of a WDTC [3]. Its clinicopathological features are distinct from that of an ATC. Due to the relative rareness of the condition and the heterogeneity of diagnostic criteria, studies on PDTC are limited. Patients with PDTC tend to have adverse clinicopathological features and often present with advanced locoregional disease and distant metastases. PDTC tends to have a high frequency of lymph node spread and the frequency of lymph nodal metastases can be as high as 50–85% of cases with distant metastases occurring in up to 85% of cases [38,39]. The incidence of lateral cervical lymph node involvement at the time of diagnosis is more frequent in patients older than 70 years [40].

The management of PDTC has not been standardized due to the rarity of the disease and heterogeneity of inclusion criteria. Surgery with removal of all macroscopic disease can achieve satisfactory locoregional control. Central and/or lateral neck dissection should be performed if there is clinical or radiological evidence of pathological lymph nodes [3]. Some authors point out that surgical intervention in operable cases involves complete central and lateral cervical lymphadenectomy [40], although this is not routinely recommended by most experts in the absence of pathologic neck disease in the respective compartment. When performing thyroidectomy, concurrent central dissection can be considered to avoid having to return later in the previously dissected thyroid bed (reducing the risk of injury to the recurrent laryngeal nerve and injury to the parathyroid glands). However, lymph node dissection does not seem to have any statistically significant impact on survival [41]. Indications and efficacy of adjuvant treatment PDTC are controversial. In patients with lymph node involvement, particularly where there is significant extranodal extension post-operative external beam radiotherapy (RT) may be indicated in an attempt to reduce the high risk of locoregional recurrence [42]. Adjuvant radio iodine (RAI) may be considered only in a subset of patients owing to variable levels of iodine uptake, although the exact number of responders is unclear.

The prognosis for these patients, when complete clearance of all gross disease is achieved, is favorable with a 5-year locoregional control of 81% [43]. However, most patients with PDTC who die of the disease do so because of distant metastases [3].

### 4.2. Anaplastic Thyroid Carcinoma

**Figure 2:**
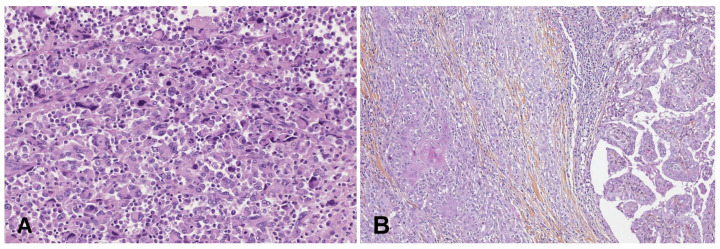
(**A**) ATC pleomorphic variant: atypical cells with abundant inflammatory infiltrate (×400 magnification). (**B**) Tall cell papillary carcinoma (right) transformated in ATC squamoid variant (left) (×100 magnification).

ATCs are undifferentiated tumors of the thyroid follicular epithelium (Figure 2). The so-called squamous cell carcinoma of the thyroid is likely a form of ATC, with similar histological appearance and biological behavior. They are extremely aggressive tumors with the mortality rate reaching 100% [44]. The reported median survival is of only 5 months, with the median 1-year survival of 20% [45]. Nonetheless, developments in the treatment of patients with the use of targeted therapies and immunotherapy appear to be associated with a significant increase in survival. In the largest single institution ATC-cohort published to date, Maniakas et al. [46] cite routine and rapid molecular testing for actionable oncogenic variants and fusions, targeted therapy, immunotherapy, and neo-adjuvant therapy prior to surgery as factors leading to a 1-year survival of 59%, with a 1.31-year median survival.

The best opportunity for a cure of ATC is comprehensive surgery, essentially one that effectively removes all clinical disease in the neck including the thyroid gland. However, due to the aggressiveness of the tumor and the fact that most patients present with masses infiltrating nearby vital cervical structures (cervical viscera), surgery is generally not indicated given the potential morbidity and poor prognosis. Indeed, the majority of ATC successfully treated surgically are in fact only formally diagnosed on post-operative histopathology. Fortunately, new targeted treatment options are emerging with promising results, after which some of the inoperable patients treated with these options become surgical candidates again [47].

In considering surgical treatment, it is important to determine the extent of the disease and the possibility of complete resection. Total or near-total thyroidectomy would only be considered in patients with resectable tumors that can be removed with acceptable morbidity. After surgery, treatment with adjuvant (chemo) RT would be appropriate. For the most advanced and unresectable disease, which is the most common situation, treatment should be individualized according to the patient’s general medical condition and care goals [48].

Nearly all patients with ATC present with a thyroid mass, and regional spread is clinically apparent at the time of diagnosis in about 50% of cases [49]. ATC has a predictable ability to grow into adjacent structures in the neck as well as spread into the lymph nodes of the neck, especially in the central compartment. The lymph nodes of the central compartment should be thoroughly assessed during the work-up procedure either with high-quality ultrasound or computed tomography. These lymph nodes are commonly involved in ATC. Nevertheless, American Thyroid Association (ATA) Guidelines for Management of Patients with Anaplastic Thyroid Cancer, published in 2021, states that prophylactic central or lateral neck node dissection in patients with ATC is not indicated, but every attempt should be made to remove clinically apparent disease [48].

If lymph node metastases at the central compartment are present, a unilateral or bilateral therapeutic lymph node dissection should be considered when the thyroid gland is removed [48]. However, the presence of enlarged lateral lymph nodes does not necessarily indicate ATC having spread, although histological confirmation should not delay surgery for operable patients and a diagnosis by an experienced radiologist would be sufficient. In ATC, neck dissection is more commonly seen as a method of three dimensionally “getting around the cancer” more often than just removing the lymph nodes themselves. Intraoperative decisions should be based on the pre-operative imaging as well as the intraoperative findings, particularly as this relates to the recurrent laryngeal nerves and their function. A neck dissection including levels II, III, IV, and V, in untreated patients, should only be performed when the spread of ATC to lymph nodes on the side of the neck has been confirmed [48].

Importantly, approximately 35% of ATC is associated with the BRAF V600E mutations. Given the dramatic response of BRAF-mutated ATC to BRAF/MEK inhibitor, all patients with stage IVB or stage IVC ATC should be quickly tested for this mutation prior to initiation of treatment including surgery (unless there is a need for an urgent surgical airway). BRAF immunohistochemistry testing can be performed in approximately 48–72 h in many centers, and the presence of this mutation will often shift the treatment paradigm to upfront systemic therapy (BRAF/MEK inhibitor). Under such circumstances, neo-adjuvant treatment with dabrafenib plus trametinib may be indicated to improve the chances of complete tumor resection [46,47,50]. This is particularly relevant in surgically fit patients who harbor the mutation and who, on pre-operative imaging, have areas which preclude gross macroscopic resection (R2 interface). In such patients, neo-adjuvant treatment may result in tumor response significant enough to warrant later definitive surgery with adjuvant therapy thereafter. 

### 4.3. Medullary Thyroid Carcinoma 

**Figure 3:**
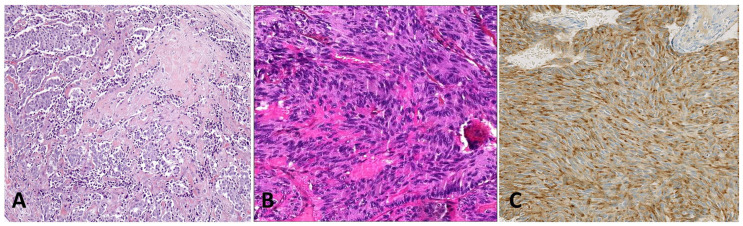
(**A**) MTC with amyloid stroma (×100 magnification). (**B**) MTC spindle cell variant (×400 magnification). (**C**) Calcitonin expression confirmed the diagnosis of MTC and ruled out a mesenchymal tumor (×200 magnification).

MTC is a neuroendocrine carcinoma that arises from thyroid parafollicular C cells and accounts for only 1–2% of thyroid cancer [51] (Figure 3). About 75% of cases are reported to be sporadic, without a known genetic cause. However, about 10% of patients presenting with suspected sporadic MTC may be carriers of germline RET mutations. About 25% of cases are reported to be familial and occur as an isolated condition (familial MTC) or as part of multiple endocrine neoplasia (MEN) type 2A or 2B syndrome.

While sporadic MTC usually presents with a solitary thyroid nodule, a significant proportion of patients may initially present with lymphadenopathy because of the high propensity for early metastatic spread to the cervical lymph nodes. Central compartment lymph node metastases are observed in approximately 50% of cases, both sporadic and hereditary. The pattern of metastatic distribution in the neck is variable and is not related to the size of the thyroid tumor. Contralateral central lymph node metastases are observed including patients with small primary tumors [52]. The superior pole location of the parafollicular C cells from which tumors are derived increases the incidence of lateral lymph node metastases compared with WDTC. Approximately 30–40% of MTC patients present with lateral lymph node metastasis at diagnosis [53]. The presence of lateral lymph nodes predicts higher tumor invasiveness, and increased risk of recurrence and death in this patient population [54].

The ATA guidelines for the management of medullary thyroid cancer have stated that patients with MTC can be treated by complete primary tumor resection and cervical lymph node dissection [51,55]. However, the extent of primary surgery regarding cervical lymph node dissection is controversial. Some authors advocate aggressive treatment of cervical nodes or prophylactic neck dissection to improve post-surgical biochemical response rates, while others advocate conservative treatment because of the possibility of complications and unknown benefits [56,57]. If cervical dissection is warranted, the extent of the dissection depends on the findings on a pre-operative ultrasound. 

In patients with MTC without evidence of cervical lymph node involvement on pre-operative ultrasound, bilateral prophylactic central compartment lymph node dissection is indicated [12,51]. Machens et al. [58] suggested that elective prophylactic central neck dissection was not necessary in patients with a small intrathyroidal MTC with a pre-operative calcitonin <20 pg/mL, as metastatic lymph nodes are extremely rare in these circumstances. It has not reached consensus on the need for lateral dissection. Most authors discourage prophylactic lateral dissections if there is no evidence of metastases on pre-operative ultrasound [12,57,59]. The rate of biochemical response has been found to be similar between observation and elective dissection of lateral neck compartments [59]. Pena et al. [59] compared the outcomes of a group of patients with MTC without clinical lateral neck metastases who underwent elective lateral neck dissection with a group who underwent observation. They observed that there was no significant difference in locoregional control, biochemical cure rate, and overall survival between both groups. Others favor using pre-operative calcitonin values to indicate the need for an elective neck dissection, recommending ipsilateral central and ipsilateral lateral prophylactic neck dissections for patients with baseline serum calcitonin values > 20 pg/mL and prophylactic dissection of contralateral lateral neck compartments not involved for serum calcitonin > 200 pg/mL [51,58]. Machens et al. [60] carried out a study on the frequency of lateral lymph node metastasis. They found ipsilateral lateral lymph node metastases in 10% of patients with no central node involvement, 77% of patients with 1 to 3 central nodes, and 98% of patients with more than 4 central nodes; contralateral lateral lymph node metastases were found in 5% of patients without central nodes, 38% of patients with 1 to 9 central nodes, and 77% with more than 9 central nodes. Nevertheless, the need to perform an elective lateral dissection remains controversial.

For patients with locoregionally advanced RET-mutated MTC, including patients with invasive nodal disease, a novel strategy of neo-adjuvant RET-specific inhibitor prior to surgical resection has been reported [61], and this approach continues to be studied in an ongoing clinical trial (NCT04759911) [62]. 

In conclusion, in patients with pre-operative evidence of lateral cervical adenopathy, bilateral dissection of the central and affected lateral compartments is indicated [51]. In the case of MTC, it is necessary to emphasize the need to dissect sublevel Vb as part of an elective dissection, even more so compared to WDTC. However, it should be noted that long-term biochemical cure rates in patients with metastatic adenopathy are low.

### 4.4. Mucoepidermoid Carcinoma 

**Figure 4:**
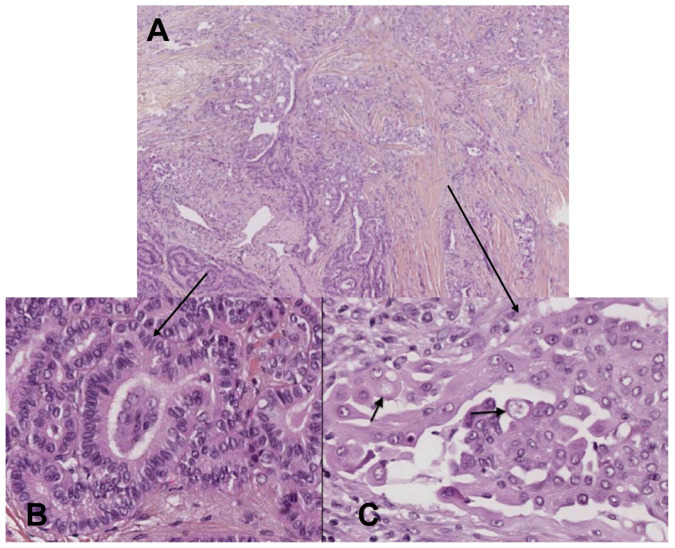
Composite MEC and columnar cell variant of papillary carcinoma. (**A**) Variable architecture: papillary, follicular, solid, and cribriform areas intermingled (×100 magnification). (**B**) Component of columnar cell variant of papillary carcinoma: papillary and follicular areas, columnar stratified neoplastic cells with an abundant cytoplasm and a hyperchromatic nucleus (×400 magnification). (**C**) Component of MEC: cribriform area, large round to polygonal tumor cells with abundant eosinophilic cytoplasm intimately admixed with few tumor cells containing clear intracytoplasmic vacuoles of mucin (arrows) (×400 magnification).

MEC is a carcinoma of unknown histogenesis classified as low, intermediate, or high grade according to the histologic features, although most cases are considered to be low grade (Figure 4). Typically, MEC arises in the salivary glands [63] but has also been observed in other organs such as the glandular structures of the larynx, esophagus, and breast. Thyroid MECs are extremely rare with less than 0.5% of all thyroid malignancies [64]. Since the first description by Rhatigan et al. [65], just over 45 cases have been reported in the literature [66].

Patients are usually asymptomatic and the tumor usually presents as a unilateral cold nodule in ultrasound. Up to 15% of the cases reported have demonstrated extrathyroidal extension and approximately 42% of the patients have lymph node metastases in the central compartment (level VI) at the time of diagnosis [66]. This high rate of lymph node metastasis could be due to MEC arising from metaplastic de-differentiation of pre-existing WDTCs (papillary and follicular thyroid cancer) [67]. 

The treatment of choice is surgery and consists of total thyroidectomy [68]. Nevertheless, a few selected patients have been managed successfully by hemithyroidectomy as these tumors are not RAI-avid tumors [69]. The role of prophylactic central neck node dissection is controversial [67]. Given the high number of metastases observed in the described cases, prophylactic central neck dissection is advised. If positive lymph nodes are observed, there is no consensus to indicate lateral neck dissection. RT and chemotherapy have been used to treat these tumors but there is still debate about adjuvant therapy [67]. As in the case of differentiated carcinomas, and based on their possible histological relationship, targeted adjuvant therapies are incorporated into the treatment [67].

Most of the reported patients show a good outcome and cases of disease-free survival of more than 10 years have been reported. Nevertheless, some with a high-grade tumor expressing adverse features have had a poor outcome [66].

### 4.5. Malignant Peripheral Nerve Sheath Tumors (MPNSTs) 

**Figure 5:**
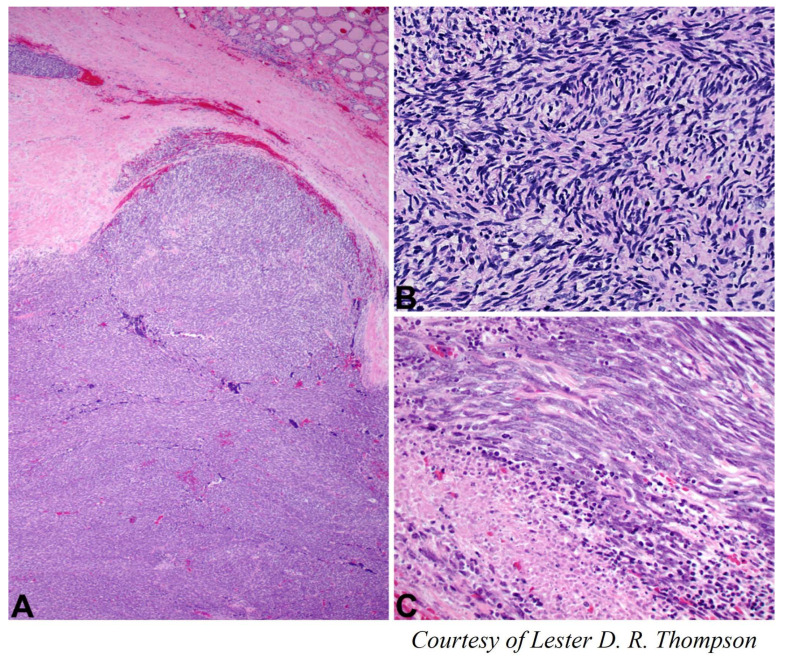
MPNST with a peritheliomatous growth pattern of the atypical spindle-cell neoplasm, showing buckled nuclei (**A** (×200 magnification), **B** (×400 magnification)). Focus of necrosis (×200 magnification) (**C**).

Peripheral nerve sheath tumors can be benign or malignant. MPNST, or malignant “Triton” tumor, is a sarcoma of the cells that form the sheath that covers peripheral nerves (Figure 5). Criteria for malignancy are the presence of nuclear polymorphism, necrosis, and increased mitotic index [70]. This tumor can grow in the soft tissues of the body including the thyroid gland. MPNST is an invasive tumor, grows quickly, and can spread to other parts of the body.

Primary MPNSTs of the thyroid gland are rare, accounting for <0.02% of all thyroid tumors worldwide [71,72]. Primary MPNST may arise within the thyroid parenchyma or in the adjacent thyroid capsule; the number of reported cases in the literature is less than 30 [73]. Thyroid MPNST usually presents as a gradually growing firm thyroid mass with progressive enlargement, with non-specific symptoms and signs, which makes pre-operative diagnosis difficult [74]. Differential diagnosis includes spindle cell tumors, Riedel thyroiditis, and ATC, as well as soft tissue tumors occurring in the thyroid gland or adjacent tissues [75]. MPNSTs can obliterate the thyroid parenchyma in a fascicular growth pattern; they are characterized by neural-like cells, increased cellularity, increased mitotic activity, and focal necrosis. Immunohistochemical staining for S-100, CD34, and vimentin can be helpful in confirming the diagnosis.

Because of the high rate of local recurrence, aggressive surgical resection including total thyroidectomy or extended total thyroidectomy when extrathyroidal extension is observed (e.g., larynx, trachea, …) to achieve wide free margins, with adjuvant radiation therapy and eventual chemotherapy currently being the mainstay of treatment of these tumors [76]. However, irrespective of aggressive adjuvant therapies, a fatal outcome is frequently observed [75]. In cases of extensive disease where complete excision is not possible, surgical debulking may be indicated to relieve compression of the airway and great vessels. Radiation therapy is known to reduce local recurrence rates but not the rate of distant metastasis [76]. Because of the low tendency of these tumors to metastasize to the cervical lymph nodes, neck dissection seems to be sonly indicated in case with clinically positive nodes.

Given the small number of cases described, it is difficult to predict the behavior of the tumor. However, most reported patients have died of their disease.

### 4.6. Angiosarcoma

**Figure 6:**
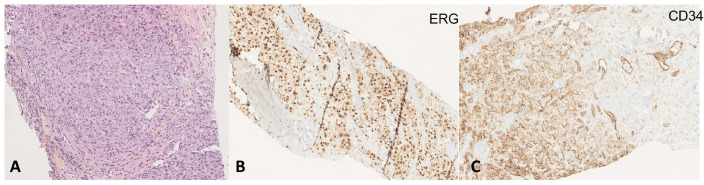
(**A**) Angiosarcoma with areas of sheet-like growth of epithelioid large cells and some vascular cavities (×100 magnification)**.** ERG (×100 magnification) (**B**) and CD34 expression (**C**) confirmed the diagnosis of angiosarcoma and ruled out an ATC (×100 magnification).

Angiosarcoma is an uncommon soft tissue sarcoma [77] (Figure 6). According to a systematic review by De Felice et al., by 2018 only 61 patients had been described in the literature [78]. Most of them were isolated cases and the largest published series comprised 12 cases [79].

Thyroid angiosarcomas is the most common of the mesenchymal thyroid tumors [80]. They usually occur in the context of long-standing multinodular goiters [81,82], mainly in the alpine region although they have been described elsewhere [83,84]. While some authors have considered them as an anaplastic or undifferentiated carcinoma with angiomatoid characteristics [85,86,87], there is now broad consensus that angiosarcomas arise from endothelial cells, characterized by positivity for the CD31 marker and erythroblast transformation-specific related gene (ERG) [77,85,88,89,90]. Clinical and radiological features are nonspecific. These tumors are highly aggressive malignant vascular tumors, characterized by a severe local course and rapid metastatic dissemination and are associated with a dismal prognosis [80,91,92,93]. Most of these tumors appear as painless, poorly encapsulated, infiltrative masses and patients present with compression symptoms including a lesion on the neck, dyspnea, hoarseness, and dysphagia due to the rapid growth. However, in many patients the definitive diagnosis is achieved only after thyroidectomy [94]. As with most sarcomas if the diagnosis is known or suspected in advance, consultation with medical and radiation oncology should be in advance, as these patients are often best managed with systemic therapy prior to surgery.

There is no established consensus on the optimal treatment approach of thyroid angiosarcoma due to its rarity [78]. In resectable tumors, aggressive surgical resection to achieve local control remains the cornerstone of primary treatment. However, most tumors are not amenable to wide local excision with generous margins of normal tissue due to anatomic constraints. Doxorubicin-based adjuvant chemotherapy for systemic control and RT for local control has been widely accepted. Local recurrences and early systemic metastases are frequent, even after complete removal [81,95].

Although the development of cervical metastases is very common, in most cases of angiosarcoma described in the literature, prophylactic treatment of the neck has not been performed [94,95,96,97]. In cases where lymph node metastases are observed at diagnosis, a bilateral central and/or lateral cervical dissection should be carried out [94].

It is important to take this tumor type into account when dealing with a thyroid tumor with aggressive histological features [85]. A correct diagnosis is important to increase early diagnosis, adequate treatment, and chances of survival.

### 4.7. Leiomyosarcoma 

**Figure 7:**
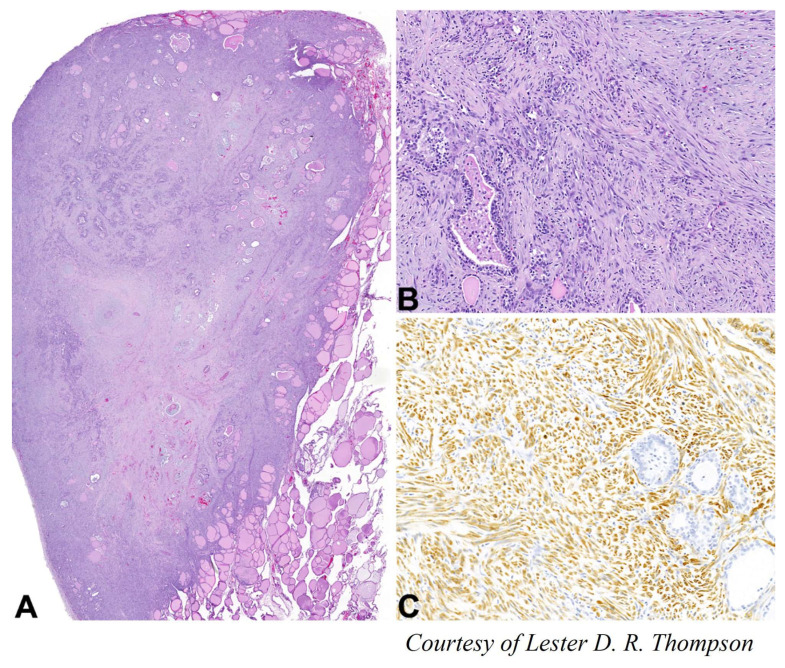
(**A**) Large destructive leiomyosarcoma within the thyroid gland (×200 magnification). (**B**) The neoplastic cells are spindled, showing an interlacing fascicular architecture (×100 magnification). (**C**) The neoplastic cells show a strong and diffuse cytoplasmic reaction with smooth muscle actin (×100 magnification).

Primary thyroid leiomyosarcomas are exceedingly rare with less than 40 cases reported in the literature [98,99,100,101] (Figure 7). Leiomyosarcoma metastases in the thyroid gland are even rarer [99]. As the thyroid gland is highly vascularized, leiomyosarcomas are considered to originate in the smooth muscles of the blood vessels [102].

These tumors usually occur in elderly people and are not related to previous thyroid lesions or radiation exposure [98,103]. They tend to present as a rapidly growing painless thyroid mass that can cause compressive symptoms, dysphagia, and dysphonia [98]. Pre-operative diagnosis can be challenging and must be differentiated from ATC, other sarcomas, and metastases [104,105,106,107]. There are no imaging characteristics or tumor markers that allow a pre-operative diagnosis, and all patients have been diagnosed following surgical resection.

Due to the limited experience with these rare tumors, there are no evidence-based treatment protocols. If radical resection is possible, surgery is the treatment of choice [102,104,108]. The surgical approaches reported in the literature vary between thyroid lobectomy to total thyroidectomy plus therapeutic modified radical neck dissection [104,109,110,111,112]. Although some authors suggest radical surgery in order to obtain local control of the disease, these tumors often show rapid infiltration to nearby structures and typically develop distant metastases. Chemotherapy and radiotherapy, as well as the use of targeted therapies, do not seem to improve outcomes [98], although some authors point to the possibility of adjuvant chemotherapy [109]. Importantly, when the diagnosis is suspected or known upfront, there should be multidisciplinary discussion prior to surgery as neo-adjuvant chemotherapy may be recommended.

### 4.8. Primary Malignant Thyroid Teratoma or Thyroblastoma

**Figure 8:**
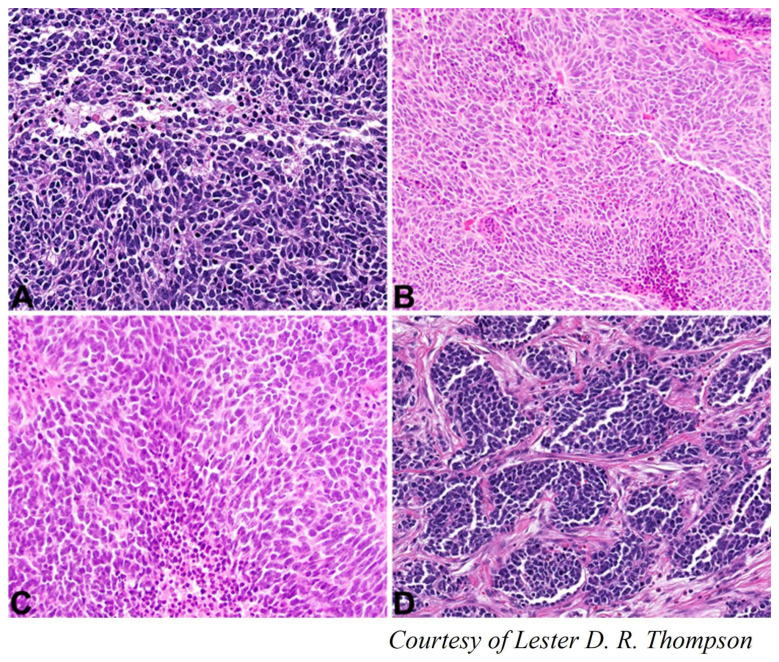
Primary Malignant Thyroid Teratoma or Thyroblastoma. Primitive small round to oval cells, disposed into irregularly communicating solid aggregates and sheets with foci of necrosis (×200 magnification) (**A**–**C**). Nests of small round-to-oval cells intermixed with primitive spindle stromal cells (×200 magnification) (**D**).

Teratoma is defined as a neoplasm that consists of tissue derived from more than one primitive germ cell layer, at least some of which is foreign to the anatomic site in which it occurs. Cervical teratomas are rare, and even more rare for those arising in the thyroid gland [113]. Currently less than 50 cases have been published [7]. As with teratomas originating in the gonads, thyroid teratomas can be divided into 3 types depending on the presence and proportion of the immature component: benign (mature), immature, or malignant. Malignant teratoma is now correctly called thyroblastoma [114] (Figure 8). The presence and proportion of immaturity influences the outcomes [115]. Thyroid teratomas can be congenital; these are increasingly diagnosed in utero and then resected in the immediate postnatal period, after securing the airway. These are mainly immature teratomas [116]. Malignant cervical teratomas usually occur in adults [115]. Malignant thyroid teratomas are characterized by recurrent DICER1 mutations, making them a distinct entity [117].

Malignant thyroid teratomas usually begin as a thyroid mass accompanied by lymphadenopathy. These tumors frequently harbor in the lungs [113,118].

These tumors should be managed aggressively to achieve best results. Surgical excision is the treatment of choice. Neo-adjuvant chemotherapy can reduce the size of the primary tumor and metastases [7]. In case of lateral cervical adenopathies, dissection of the central compartment and lateral dissection of the affected side would be indicated. Adjuvant therapy would be reserved for the malignant cases [118]. 

Local tumor recurrence or distant metastatic disease determines patient prognosis [118].

## 5. Conclusions

The rich lymphatic supply of the thyroid gland, coupled with the high propensity for the development of lymph node metastases in malignant thyroid tumors, warrants the head and neck surgeon to be familiar with the tumor-specific indications and techniques of regional lymph node dissection.

Nodal metastases can negatively affect prognosis and locoregional control in all forms of thyroid malignancies and should be considered and treated to reduce regional recurrence rates, with minimal morbidity.

In non-WTDC, the rate of nodal involvement is variable and depends on the histology of the tumor. While in MTC, PDTC, and ATC, with high frequency of lymph node metastases, prophylactic central compartment dissection is generally necessary. For mucoepidermoid carcinomas, malignant peripheral nerve sheath tumors, sarcomas, and malignant thyroid teratomas, central compartment dissection is less often necessary, as clinical lymph node involvement is less common.

Within non-WDTC there is an established consensus on the treatment of ATC, PDTC, and MTC. However, the management of other rarer tumors needs to be individualized. For the management of neck nodes for these rare tumors, the evidence is lacking due to a paucity in numbers. Clinicians must consider both the aggressiveness of the histology and the anatomical distribution of disease. 

All these cases merit multidisciplinary involvement to discuss the potential for pre-operative systemic therapy and post-operative adjuvant therapies. By approaching patients with a multidisciplinary individualized approach, a tailored treatment plan can be devised. In all cases, tumor and patient-related factors should be considered to minimize morbidity and maximize clinical impact on a rare and diverse group of diseases.
